# Fragmentation of Ventricular Extrasystoles: A Potential New Electrocardiographic Window to Uncover Patients at Risk

**DOI:** 10.7759/cureus.13748

**Published:** 2021-03-07

**Authors:** Islam M Shatla, Yasser Sammour, Mahmoud El Iskandarani, Angel López-Candales

**Affiliations:** 1 Internal Medicine, University of Missouri Kansas City School of Medicine, Kansas City, USA; 2 Internal Medicine, East Tennessee State University, Johnson City, USA; 3 Cardiovascular Medicine, University of Missouri Kansas City, Kansas City, USA

**Keywords:** qrs fragmentation, premature ventricular contractions, coronary artery disease

## Abstract

Fragmented QRS (fQRS) is a marker of conduction block due to myocardial scar that presents in electrocardiography (ECG) as an additional one or more R wave (R’) or notching in the S wave nadir in contiguous leads. However, fQRS description on premature ventricular contractions (PVCs) has not been previously described. We describe a case of a 67-year-old male with a past medical history of prediabetes, hypertension and coronary artery disease who presented after an ophthalmic procedure with asymptomatic PVCs and episodes of bigeminy. Initial ECG showed an isolated fQRS in V2.

However, during PVCs significant extrasystoles fragmentation was seen in other coronary territories. Upon reviewing his most recent cardiac catheterization, it showed a 40% ostial and 70% distal left anterior descending stenosis with a mid-segment patent stent, 95% first diagonal stenosis and totally occluded proximal right coronary artery. Identification of diffuse fQRS known to be associated with myocardial scar, sustained arrhythmic events and sudden cardiac death, particularly when seen in the inferior leads, became extremely relevant in our patient. We noted that ejection fraction reduction from 52% to 34% on his last coronary intervention was crucial to decide if an implantable cardioverter-defibrillator would be needed. PVC fragmentation might be a new ECG marker that could uncover both scar and arrhythmia potential in patients at risk of adverse cardiac events.

## Introduction

We describe a case in which QRS fragmentation was only identified on premature ventricular contractions (PVCs) seen on the surface electrocardiogram (ECG) in a patient with prior history of coronary artery disease (CAD). The importance of this electrocardiographic finding is discussed along with the possibility for this new finding to be considered as a potential additional tool for recognizing patients that might be at risk of adverse cardiac events.

## Case presentation

A 67-year-old male patient with past medical history of CAD, hypertension, prediabetes, obesity, and chronic back as well as hip pain presented to the emergency department after an ophthalmic procedure (Phaco and intraocular lens [IOL] implant) with asymptomatic PVCs with the intermittent occurrence of bigeminy. At the time, the patient was free of any chest pain, shortness of breath, dizziness, or lightheadedness; he had no signs or symptoms of decompensated heart failure.

**Figure 1:**
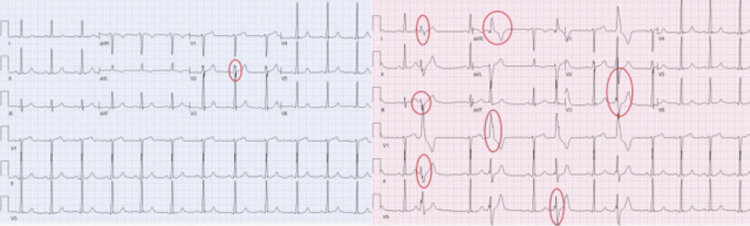
(a) Showing fragmented QRS (fQRS) in V2 without premature ventricular contractions (PVCs), (b) During PVCs significant fragmentation of the extrasystoles is noted in other coronary territories.

His ECG showed normal sinus rhythm with the presence of fragmented QRS (fQRS) complexes in lead V2 in the absence of PVCs (Figure 1A). However, with the occurrence of PVCs, there was significant fragmentation of the extrasystoles seen in other coronary territories (Figure 1B). 

Review of his most recent cardiac history revealed that this patient had a coronary angiogram in 2014 showing ostial left anterior descending (LAD) artery 50% stenosis, mid LAD 70% stenosis, first diagonal 95% stenosis and totally occluded right coronary artery (RCA). He had mid LAD stent placed at this time. In 2020, he presented with chest pain on exertion and the decision was made at that time to proceed with a myocardial perfusion nuclear imaging study which revealed 21% of the left ventricular (LV) mass was ischemic. His resting left ventricular ejection fraction (LVEF) was 34% with global hypokinesis but during stress his LVEF fell to 25% with worsening wall motion abnormality noted anteriorly with an attenuated global myocardial blood flow reserve of 1.24. In view of these results, another coronary angiogram was then performed showing a patent mid LAD stent with a distal LAD 70% stenosis, a 95% first diagonal stenosis, and a totally occluded RCA with left circumflex artery (LCX) minor irregularities. Percutaneous coronary intervention (PCI) to both distal LAD and first diagonal branch were performed resulting in resolution of symptoms and overall clinical improvement. LVEF improved after revascularization from 34% to 40% as well.

## Discussion

The fragmentation of QRS is defined as the presence of an additional one or more R wave (R’), or notching in the nadir of the S wave in contiguous leads on ECG that does not fit into left or right bundle branch block (LBBB, RBBB) morphology [1]. In the case of wide complex QRS such as LBBB, RBBB, PVCs or paced QRS, fQRS can be defined as: (A) Fragmentation greater than two notches in the R or S wave in ≥2 contiguous leads; (B) Presence of ≥4 spikes in one or ≥8 spikes in all of the leads V1, V2 and V3 [2,3]. fQRS represents abnormal ventricular depolarization that could be related to myocardial fibrosis and scar formation [4]. 

Normal ventricular depolarization starts by interventricular septum depolarization from left to right followed by left and right ventricular wall depolarization which occurs simultaneously but in opposite directions [5]. In the case of RBBB or LBBB, lack of simultaneously ventricular contractions leads to early depolarization of the opposite ventricle wall causing characteristic ECG changes.

Electrocardiographically, RBBB appears as wide QRS (>120 msec) with RSR` in leads V1 and V2 and S wave greater duration than the R wave in lead I and V6, whereas LBBB appears as wide QRS (>120 msec) with broad R wave in lateral leads lead I, AVL, V5 and V6 and S wave greater duration than the R wave in V1 and V2 [6]. fQRS is thought to be related to alteration in normal ventricular depolarization.

Even when fragmentation of PVCs (fPVC) have been described and defined as the presence of more than two R′ or more than two notches in the S waves in two contiguous leads or PVCs with only two notches in the R wave but were >40 ms apart and present in two contiguous leads [7]; their association as characterized for fQRS has been less well characterized.

fQRS was studied in a wide variety of cardiac conditions. It is considered as a marker for different cardiovascular diseases including CAD, arrhythmia, non-ischemic cardiomyopathy (NICM), infiltrative cardiac diseases and congenital heart disease [8]. In patients presenting with acute coronary syndrome, the presence of fQRS suggests myocardial scar formation with high sensitivity and specificity [1]. More specifically in patients with ST-elevation myocardial infarction, the presence of fQRS was associated with failure of thrombolytic or PCI [9]. Further, higher frequency of fQRS has been linked with slow coronary flow [10]. 

In the setting of NICM, previous studies demonstrated increased all-cause mortality and ventricular tachyarrhythmias in NICM patients with fQRS compared with patients without fragmentation [11]. Fragmentation can also predict the occurrence of ventricular arrhythmias in NICM patients who received implantable cardioverter defibrillators (ICD) for primary or secondary prevention [12]. In patients with NICM, the number of ECG leads with fQRS correlates inversely with LVEF, however there was no association with mortality [13]. Resolution of fQRS after cardiac resynchronization therapy (CRT) implantation is associated with favorable response to CRT [14].

Moreover, QRS fragmentation was found in 85% of patients with arrhythmogenic right ventricular dysplasia (ARVD) in comparison with only 4% of those without ARVD. This study demonstrated that QRS fragmentation in ARVD has a high diagnostic value similar to epsilon potentials in ARVD [15].

## Conclusions

The uniqueness of our case is that it highlights the occurrence of PVCs fragmentation above and beyond what has been described about fragmentation of QRS complexes on the surface ECG. Specifically, even when a fQRS complex was initially seen in one lead (V2), probably related to previous PCI procedures in that LAD territory, occurrence of PVCs unmasked the occurrence of additional fragmentation seen in other coronary territories. Future studies are now needed to investigate the prognostic utility of fQRS as a potentially helpful ECG marker that may offer insight into underlying scar burden and potential arrhythmia risk in patients with CAD.
